# From Real-World Research to Real-World Decisions: Insights from ICHR-26 at Rehman Medical Institute Peshawar

**DOI:** 10.12669/pjms.42.6.19607

**Published:** 2026-06

**Authors:** Seelay Srak Rehman

**Affiliations:** Seelay Srak Rehman, Director RCN, Rehman College of Nursing, Hayatabad, Peshawar

**Keywords:** Translational research, real-world decision-making, conference dissemination, institutional visibility, research culture, ICHR-26

## Abstract

Fourth International Conference on Health Research (ICHR-26), hosted by Rehman Medical Institute (RMI), brought into focus an important challenge within the current health research landscape, namely the growing disconnect between research production and its practical applicability in real-world decision-making. The conference emphasized that the problem is not a lack of research, but the large volume of studies that fail to guide practice, education, and policy due to limited contextual relevance and weak translation into action.

The conference also highlighted key factors to help bridge this gap, including stronger academic platforms for sharing knowledge, and the contribution of practice-based disciplines, particularly nursing, in linking research to real healthcare settings. It also proposed a practical framework to strengthen the link between research and its real-world use, built on three key pillars: contextual relevance, strategic dissemination, and translation into practice. Together, these elements support the production of research that is scientifically sound, meaningful to local healthcare needs, and capable of informing clinical and policy decisions. In doing so, the conference reinforced RMI’s vision of promoting impact.

## INTRODUCTION

Health research is increasingly evaluated not only by its academic merit but by its ability to improve patient care, guide policy decisions, and strengthen healthcare systems. Despite a growing volume of scientific output, a persistent gap remains between producing research and using it in real-world healthcare. Many studies are published but fail to influence clinical practice, policy, or education because they are poorly aligned with practical needs or lack clear implementation value.^1^,^2^ ICHR-26 was organized around this challenge. Its theme, *From Real-World Research to Real-World Decisions*, reflects a shift from viewing research as an academic endpoint to using it as a tool for meaningful change in healthcare systems.

While conferences serve as important platforms for academic exchange, their impact often remains limited to attendees. The ideas discussed, debated, and refined during such gatherings must be extended into the broader academic domain to achieve their intended purpose. This necessitates the transformation of conference themes into scholarly communications that can reach a wider audience, stimulate dialogue, and influence future research directions. The present communication aims to achieve this by translating the core message of ICHR-26 into a structured academic narrative. It also serves to highlight the role of RMI in promoting research that is aligned with real-world healthcare needs.

### From Conference Theme to Institutional Impact:

The conference functioned as a multidimensional academic platform where research was explored through diverse scholarly and practical engagements. Alongside keynote lectures, plenary sessions, and panel discussions led by national and international experts, the programme included pre-conference workshops, oral and poster presentations, rapid-fire sessions, research arena activities, practice-changing case presentations, quiz competitions, innovation showcases, science cafés, and networking forums.^3^ Dedicated undergraduate and postgraduate research corners, travel grants, young investigator awards, and research seed grant opportunities created space for emerging researchers to engage meaningfully with evidence generation and application. Notably, the Editors’ Summit added an important dimension by bringing editors, reviewers, and researchers together to discuss publication standards, research integrity, and the role of scientific publishing in translating evidence into practice. Together, these activities reflected a broader framework for impactful research, grounded in contextual relevance, scholarly rigor, strategic dissemination, and practical implementation, while reinforcing RMI’s role in shaping a research culture that values not only publication, but real-world healthcare impact.

### Emerging Role of Artificial Intelligence:

Artificial Intelligence (AI) emerged as an important area of discussion, particularly for its expanding role in shaping how research is designed, written, and disseminated. Its utility in accelerating literature searches, assisting data handling, and improving manuscript structure was acknowledged as a valuable support to researchers. At the same time, concerns were raised about its unchecked use and the implications for scientific quality.

A recurring concern was the rise of manuscripts that appear technically polished yet lack conceptual strength. Well-written AI-assisted drafts may conceal weak research questions, mismatched study designs, or poorly defined outcomes, creating a false impression of scientific rigor. This places greater responsibility on educators, supervisors, and journal editors to assess the substance of research beyond its presentation.

The discussions reinforced that AI should strengthen, not replace, human reasoning. Its value lies in supporting efficiency, while the intellectual core of research which is critical thinking, methodological decisions, and interpretation, must remain the responsibility of the researcher. In this regard, the need for institutional and editorial guidance on ethical AI use was strongly emphasized to protect research credibility, originality, and practical relevance.

### Nursing and Research Culture:

Nursing emerged as a prominent and practice-driven discipline within the conference, reflecting its central role in translating evidence into frontline healthcare decisions. The dedicated parallel session on “Empowered Nursing Research for Quality, Safety, and Leadership in Care” highlighted how nursing-led inquiry directly shapes patient safety, quality improvement, leadership practices, and system efficiency. Unlike many disciplines where research remains distant from implementation, nursing research was positioned as inherently embedded within real-time clinical care, making it one of the strongest bridges between evidence and action.

This focus was further strengthened by the institutional presence of Rehman College of Nursing (RCN), a key academic arm of RMI with an established commitment to advancing nursing education and scholarship. Through research presentations, poster sessions, student competitions, and interdisciplinary scientific forums, nursing students and faculty actively contributed to the broader academic discourse, demonstrating how nursing perspectives can identify care gaps, improve patient outcomes, and inform system-level decisions. The integration of nursing into mainstream health research discussions reinforced its evolving role not merely as an implementing profession, but as a strategic contributor to shaping research priorities and strengthening evidence-based healthcare delivery.

### The Translational Gap in Health Research:

A critical issue highlighted through the theme of ICHR-26 was not merely the existence of a gap between research and decision-making, but the nature and origin of this gap within the research process itself. In many low- and middle-income settings, including Pakistan, the challenge is not the absence of research activity, but the limited usability of the research being produced.^4^

A significant proportion of studies fail to influence clinical practice or policy decisions because they are not designed with application in mind. Research questions are often poorly constructed, lacking alignment with real-world clinical or educational problems. Many studies rely on descriptive or perception-based designs even when the underlying question requires analytical or interventional approaches. As a result, the findings generated are frequently incapable of informing decisions, regardless of statistical significance.^5^

Another critical limitation lies in the absence of clearly defined and measurable outcomes. Studies often include variables that are neither operationally defined nor quantifiable, making it difficult to interpret results in a meaningful way. Without precise measurement, research cannot be translated into protocols, guidelines, or policy recommendations. This disconnect between what is studied and what can actually be acted upon remains a major barrier to translation.

Furthermore, there is a tendency to prioritize publication over purpose. Academic progression and institutional metrics continue to emphasize the number of publications rather than their impact. This has led to a growing body of literature that is methodologically weak and contextually irrelevant. In such an environment, even well-intentioned research efforts fail to contribute to healthcare improvement.

The implications of this gap are substantial. When research does not translate into decision-making, it results in missed opportunities for improving patient outcomes, inefficient use of limited resources, and stagnation in health system development. More importantly, it undermines the credibility of research as a tool for change.

Addressing this issue requires a shift from research as an academic requirement to research as a decision-making instrument. This begins with strengthening the fundamentals of research design, ensuring that questions are relevant, outcomes are measurable, and methodologies are aligned with the intended application. Only then can research move beyond theoretical contribution and begin to influence real-world decisions.

### A Framework for Translational Research:

To strengthen the link between evidence generation and healthcare decision-making, a translational framework is proposed based on three interdependent components:

1. ***Relevance to Real-World Contexts:*** Research must begin with clearly identified clinical, educational, or public health needs. Framing questions around real-world challenges ensures that study objectives remain purposeful, outcomes are meaningful, and findings are directly applicable to practice and policy.
2. ***Strategic Dissemination as Knowledge Mobilization:*** The impact of research depends on its reach. Dissemination should extend beyond publication through academic forums, interdisciplinary collaboration, and stakeholder engagement, allowing findings to be communicated in ways that are accessible, interpretable, and capable of informing decisions.
3. ***Translation into Practice and Policy:*** The ultimate value of research lies in its application. Evidence must be translated into clinical protocols, educational strategies, and policy actions through clear implementation pathways, ensuring that findings contribute to measurable improvements in healthcare systems.^6^

Together, these three components establish a practical model for translational research, shifting the focus from knowledge production alone toward relevance, usability, and real-world impact.

### The Way Forward:

To strengthen the connection between research and real-world decision-making, several priorities must be addressed which are given below:

- Strengthening research methodology training at undergraduate and postgraduate levels
- Framing research questions around real clinical, educational, and public health needs
- Prioritizing measurable and outcome-driven research designs
- Encouraging interdisciplinary collaboration and stakeholder engagement
- Strengthening institutional platforms for research dissemination and knowledge exchange
- Aligning research agendas with local healthcare priorities and system needs
- Developing clearer pathways for translating evidence into clinical practice and policy.

## CONCLUSION

By bringing together researchers, clinicians, educators, students, and editors, ICHR-26 reinforced the importance of keeping research closely aligned with real-world healthcare needs and decision-making. Beyond scientific exchange, the conference highlighted the changing landscape of health research, where emerging tools such as AI are becoming increasingly difficult to ignore. Its key contribution lies in encouraging a research culture that remains adaptive, ethically grounded, and focused on meaningful application in practice.

### *Author Information:

Seelay Srak Rehman is the Director of Rehman College of Nursing, Peshawar, With over 15 years of experience in academic leadership, she has expanded key nursing programs and strengthened faculty development. She holds a Master’s degree in International Relations and Politics from the University of Kent at Canterbury UK and is an alumna of Wolfson College, University of Cambridge UK, with a background in international media including BBC World Service, Radio France and National Geographic tv UK.
